# An Efficient Diagnosis System for Parkinson's Disease Using Kernel-Based Extreme Learning Machine with Subtractive Clustering Features Weighting Approach

**DOI:** 10.1155/2014/985789

**Published:** 2014-11-18

**Authors:** Chao Ma, Jihong Ouyang, Hui-Ling Chen, Xue-Hua Zhao

**Affiliations:** ^1^College of Computer Science and Technology, Jilin University, No. 2699, QianJin Road, Changchun 130012, China; ^2^Key Laboratory of Symbolic Computation and Knowledge Engineering of Ministry of Education, Jilin University, Changchun 130012, China; ^3^College of Physics and Electronic Information, Wenzhou University, Wenzhou 325035, China

## Abstract

A novel hybrid method named SCFW-KELM, which integrates effective subtractive clustering features weighting and a fast classifier kernel-based extreme learning machine (KELM), has been introduced for the diagnosis of PD. In the proposed method, SCFW is used as a data preprocessing tool, which aims at decreasing the variance in features of the PD dataset, in order to further improve the diagnostic accuracy of the KELM classifier. The impact of the type of kernel functions on the performance of KELM has been investigated in detail. The efficiency and effectiveness of the proposed method have been rigorously evaluated against the PD dataset in terms of classification accuracy, sensitivity, specificity, area under the receiver operating characteristic (ROC) curve (AUC), *f*-measure, and kappa statistics value. Experimental results have demonstrated that the proposed SCFW-KELM significantly outperforms SVM-based, KNN-based, and ELM-based approaches and other methods in the literature and achieved highest classification results reported so far via 10-fold cross validation scheme, with the classification accuracy of 99.49%, the sensitivity of 100%, the specificity of 99.39%, AUC of 99.69%, the *f*-measure value of 0.9964, and kappa value of 0.9867. Promisingly, the proposed method might serve as a new candidate of powerful methods for the diagnosis of PD with excellent performance.

## 1. Introduction

Parkinson's disease (PD) is one degenerative disease of the nervous system, which is characterized by a large group of neurological conditions called motor system disorders because of the loss of dopamine-producing brain cells. The main symptoms of PD are given as follows: (1) tremor or trembling in hands, arms, legs, jaw, or head, (2) rigidity or stiffness of the limbs and trunk, (3) bradykinesia or slowness of movement, (4) postural instability or impaired balance (http://www.ninds.nih.gov/research/parkinsonsweb/index.htm, last accessed: April 2012). At present, PD has an impact on about 1% of the worldwide population over the age of 50; however, this proportion is on the increase as people live longer [1]. Till now, PD has no medical treatment and some dedication is only available for relieving the symptoms of disease [2]. It is so important that we gain more of insight into the problem and improve our methods to deal with PD. Here we focus on the study based on dysphonia, which is known as a group of vocal impairment symptoms; it is reported to be one of the most significant symptoms of PD [3]. The researches have shown that about 90% of people with PD have such vocal evidence. The dysphonic indicators of PD make speech measurements as an important part of diagnosis [4]. Dysphonic measures have been proposed as a reliable tool to detect and monitor PD [5, 6].

Previous studies on the PD problem based on machine learning methods have been undertaken by various researchers. Little et al. [6] used support vector machine (SVM) classifier with Gaussian radical basis kernel function to predict PD, by means of feature selection method to reduce the feature space, and best accuracy rate of 91.4% was obtained by the proposed model. Shahbaba and Neal [7] presented a nonlinear model based on Dirichlet mixtures for the PD classification, compared with multinomial logit models, decision trees, and SVM; the classification accuracy of 87.7% was achieved by the proposed model. Das [8] used a comparative study of neural networks (NN), DMneural, regression and decision trees for the diagnosis of PD; the experiment results had shown that the NN method achieved the overall classification performance of 92.9%. Sakar and Kursun [9] used mutual information measure to combine with SVM for the diagnosis of PD and achieved the classification result of 92.75%. Psorakis et al. [10] introduced sample selection strategies and model improvements for multiclass multikernel relevance vector machines and achieved the classification accuracy of 89.47% in the PD dataset. Guo et al. [11] combined genetic programming and the expectation maximization (EM) to diagnose PD in the ordinary feature data and achieved the classification accuracy of 93.1%. Luukka [12] proposed a new method which used fuzzy entropy measures to combine with the similarity classifier to predict PD, and the mean classification of 85.03% was achieved. Li et al. [13] introduced a fuzzy-based nonlinear transformation approach together with SVM in the PD dataset; best classification accuracy of 93.47% was obtained. Ozcift and Gulten [14] combined the correlation based feature selection method with the rotation forest ensemble classifier of 30 machine learning algorithms to distinguish PD; the proposed model got best classification accuracy of 87.13%. Åström and Koker [15] achieved highest classification accuracy of 91.2% by using a parallel neural network model for PD diagnosis. Spadoto et al. [16] adopted evolutionary based method together with the optimum-path forest (OPF) classifier for PD diagnosis, and best classification accuracy of 84.01% was obtained. Polat [17] applied the fuzzy *C*-means (FCM) clustering feature weighting (FCMFW) together with the *k*-nearest neighbor classifier for detecting PD; the classification accuracy of 97.93% was obtained. Chen et al. [18] proposed a model which used the principle component analysis based feature extraction together with the fuzzy *k*-nearest neighbor method to predict PD and achieved best classification accuracy of 96.07% by the proposed model. Daliri [19] presented a chi-square distance kernel-based SVM to discriminate the subjects with PD from the healthy control subjects using gait signals, and the classification result of 91.2% was obtained. Zuo et al. [20] used a new diagnosis model based on particle swarm optimization (PSO) to strengthen the fuzzy *k*-nearest neighbor classifier for the diagnosis of PD, and the mean classification accuracy of 97.47% was achieved.

From these works, it can be seen that most of the common classifiers from machine learning community have been used for PD diagnosis. For the nonlinear classification problems, the data preprocessing methods such as feature weighting, normalization, and feature transformation could increase the performance of alone classifier algorithm. So it is obvious that the choice of an efficient feature preprocessing method and an excellent classifier is of significant importance for the PD diagnosis problem. Aiming at improving the efficiency and effectiveness of the classification performance for the diagnosis of PD, in this paper, an efficient features weighting method called subtractive clustering features weighting (SCFW) and a fast classification algorithm named kernel-based extreme learning machine (KELM) are examined. The SCFW method is used to map the features according to data distributions in dataset and transform linearly nonseparable dataset to linearly separable dataset. In this way, the similar data within each feature are prone to getting together so that the distinction between classes is increased to classify the PD datasets correctly. It is reported that SCFW method can help improve the discrimination abilities of classifiers in many applications, such as traffic accident analysis [21] and medical datasets transformation [22]. KELM is the improved version of ELM algorithm based on kernel function [23]. The advantage of KELM is that only two parameters (the penalty parameter *C* and the kernel parameter *γ*) need to be adjusted, unlike ELM which needs to specify the suitable values of weights and biases for improving the generalization performance [24]. Furthermore, KELM not only trains as fast as that of ELM, but also can achieve good generalization performance. The objective of the proposed method is to explore the performance of PD diagnosis using a two-stage hybrid modeling procedure via integrating SCFW with KELM. Firstly the proposed method adopts SCFW to construct the discriminative feature space through weighting features, and then the achieved weighted features serve as the input of the trained KELM classifier. To evaluate the performance of proposed hybrid method, classification accuracy (ACC), sensitivity, specificity, AUC, *f*-measure, and kappa statistic value have been used. Experimental results have shown that the proposed method achieves very promising results based on proper kernel function by 10-fold cross validation (CV).

The main contributions of this paper are summarized as follows.

1. It is the first time that we have proposed to integrate SCFW approach with KELM classifier to detect PD in an efficient and effective way.
2. In the proposed system, SCFW method is employed as data preprocessing tool to strengthen the discrimination between classes for further improving the distinguishing performance of KELM classifier.
3. Compared with the existing methods in previous studies, the proposed diagnostic system has achieved excellent classification results.

The rest of the paper is organized as follows.  Section 2 offers brief background knowledge on SCFW and KELM. The detailed implementations of the diagnosis system are presented in Section 3. In the next section, the detailed experiment design is described, and Section 5 gives the experiment results and discussions of the proposed method. Finally, conclusions and recommendations for future work are summarized in Section 6.

## 2. The Theoretical Background of the Related Methods

### 2.1. Subtractive Clustering Features Weighting (SCFW)

Subtractive clustering is the improved version of mountain clustering algorithm. The problem of mountain clustering is that its calculation grows exponentially with the dimension of the problem. Subtractive clustering has solved this problem using data points as the candidates for cluster centers, instead of grid points as in mountain clustering, so the calculation cost is proportional to the problem size instead of the problem dimension [25]. The subtractive clustering algorithm can be briefly summarized as follows:

**Step 1 .**

Consider a collection of *n* data points {*x*_1_, *x*_2_,…, *x*_*n*_} in *M*-dimensional space. Since each data point is a candidate for cluster center, the density measure at data point *x*_*i*_ is defined as

(1) $$\begin{array}{c} D_{i}=\overset{n}{\underset{j=1}{\sum }}\exp\left(\frac{-{\left\|x_{i}-x_{j}\right\|}^{2}}{{\left(r_{a}/2\right)}^{2}}\right), \end{array}$$

where *r*_*a*_ is a positive constant defining a neighborhood radius; it is used to determine the number of cluster centers. So, a data point will have a high density value if it has many neighboring data points. The data points outside the neighborhood radius contribute slightly to the density measure. Here, *r*_*a*_ is set to 0.5.

**Step 2 .**

After the density measure of each data point has been calculated, the data point with the highest density measure is selected as the first cluster center. Let *X*_*c*1_ be the point selected and *D*_*c*1_ the density measure. Next, the density measure for each data point *x*_*i*_ is revised as follows:

(2) $$\begin{array}{c} D_{i}=D_{i}-D_{c1}\exp\left(\frac{-{\left\|x_{i}-x_{j}\right\|}^{2}}{{\left(r_{b}/2\right)}^{2}}\right), \end{array}$$

where *r*_*b*_ is a positive constant and *r*_*b*_ = *η* · *r*_*a*_, *η* is a constant greater than 1 to avoid cluster centers being in too close proximity. In this paper, *r*_*b*_ is set to 0.8.

**Step 3 .**

After the density calculation for each data point is revised, the next cluster center *X*_*c*2_ is selected and all the density calculations for data point are revised again. The process is repeated until a sufficient number of cluster centers are generated.

**Figure 1:**
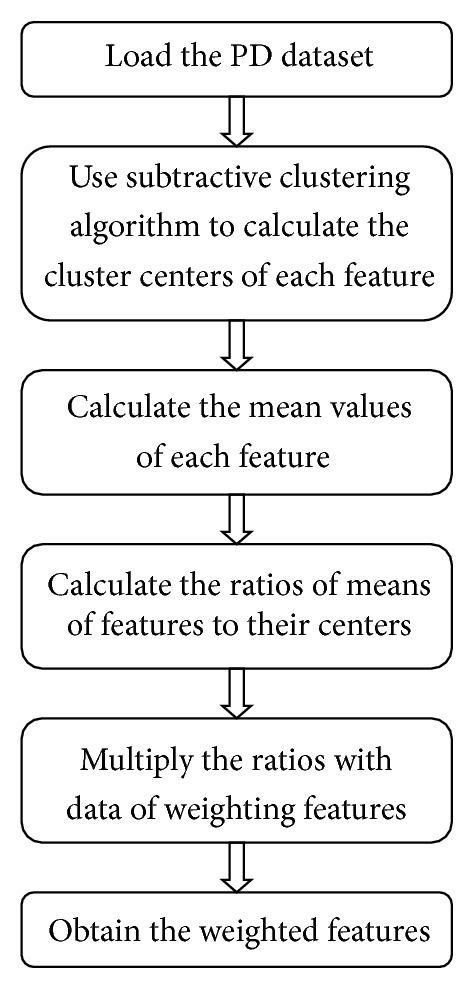
The flowchart of SCFW algorithm.

For SCFW method, firstly the cluster centers of each feature are calculated by using subtractive clustering. After calculating the centers of features, the ratios of means of features to their cluster centers are calculated and these ratios are multiplied with the data of each feature [21]. The pseudocode of SCFW method is given in Algorithm 1, and the flowchart of weighting process is shown in Figure 1.

### 2.2. Kernel-Based Extreme Learning Machine (KELM)

**Figure 2:**
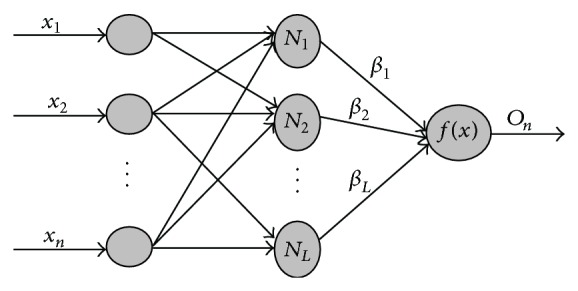
The structure of ELM.

ELM is an algorithm originally developed for training single hidden layer feed-forward neural networks (SLFNs) [26]. The essence of ELM is that parameters of hidden neurons in neural network are randomly created instead of being tuned and then fixed the nonlinearities of the network without iteration.  Figure 2 shows the structure of ELM.

For given *N* samples (**x**, **y**) having *L* hidden neurons and activation function *h*(*x*), the output function of ELM is defined as follows:

(3) $$\begin{array}{c} f\left(x\right)=\overset{L}{\underset{i=1}{\sum }}\beta_{i}h_{i}\left(x\right)=h\left(x\right)\beta , \end{array}$$

where **β** = [*β*_1_, *β*_2_,…, *β*_*L*_] is the output weight connecting hidden nodes to output nodes. **H** = {*h*_*ij*_}  (*i* = 1,…, *N* and *j* = 1,…, *L*) is the hidden layer output matrix of neural network. *h*(*x*) actually maps the data from the **d**-dimensional input space to the **L**-dimensional hidden layer feature space **H**, and thus, *h*(*x*) is indeed a feature mapping.

The determination of the output weights is calculated by the least square method:

(4) $$\begin{array}{c} \beta^{\prime }=H^{+}T, \end{array}$$

where **H** ^+^ is the Moore-Penrose generalized inverse [26] of the hidden layer output matrix **H**.

To improve the generalization capabilities of ELM in comparison with the least square solution-based ELM, Huang et al. [23] proposed kernel-based method for the design of ELM. They suggested adding a positive value 1/*C* (where *C* is a user-defined parameter) for calculating the output weights such that

(5) $$\begin{array}{c} \beta =H^{T}{\left(\frac{I}{C}+HH^{T}\right)}^{-1}T. \end{array}$$

Therefore, the output function is expressed as follows:

(6) $$\begin{array}{c} f\left(x\right)=h\beta =h\left(x\right)H^{T}{\left(\frac{I}{C}+HH^{T}\right)}^{-1}T. \end{array}$$

When the hidden feature mapping function *h*(*x*) is unknown, a kernel matrix for ELM is used according to the following equation:

(7) $$\begin{array}{c} \Omega_{\text{ELM}}=HH^{T}:\Omega_{\text{ELM}i,j}=h\left(x_{i}\right)\cdot h\left(x_{j}\right)=K\left(x_{i},x_{j}\right), \end{array}$$

where *K*(*x*_*i*_, *x*_*j*_) is a kernel function. Many kernel functions, such as linear, polynomial, and radial basis function, can be used in kernel-based ELM. Now the output function of KELM classifier can be expressed as

(8) $$\begin{array}{c} f\left(x\right)={\left[\begin{array}{c} K\left(x,x_{1}\right)\\ \vdots \\ K\left(x,x_{N}\right) \end{array}\right]}^{T}{\left(\frac{I}{C}+\Omega_{\text{ELM}}\right)}^{-1}T. \end{array}$$

## 3. The Proposed SCFW-KELM Diagnosis System

**Figure 3:**
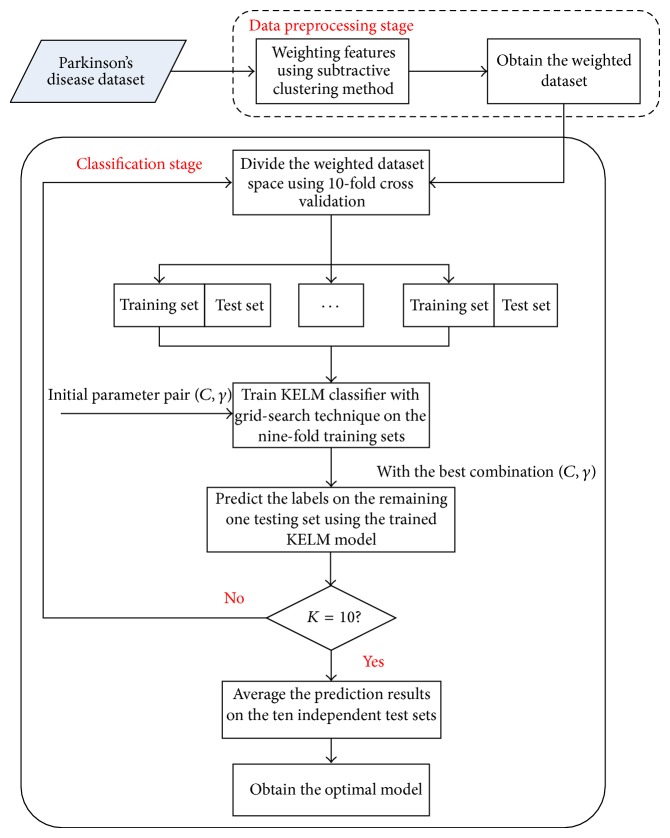
The overall procedure of the proposed hybrid diagnosis system.

This work proposes a novel hybrid method for PD diagnosis. The proposed model is comprised of two stages as shown in Figure 3. In the first stage, SCFW algorithm is firstly applied to preprocess data in the PD dataset. The purpose of this method is to map the features according to their distributions in dataset and to transform from linearly nonseparable space to linearly separable one. With this method, similar data in the same feature are gathered, which will substantially help improve the discrimination ability of classifiers. In the next stage, KELM is evaluated on the weighted feature space with different types of activation functions to perform the classification. Finally, the best parameters and the suitable activation function are obtained based on the performance analysis. The detailed pseudocode of the hybrid method is given in Algorithm 2.

## 4. Experimental Design

### 4.1. Data Description

In this section, we have performed the experiments in the PD dataset taken from University of California Irvine (UCI) machine learning repository (http://archive.ics.uci.edu/ml/datasets/Parkinson, last accessed: January 2013). It was created by Max Little of the University of Oxford, in collaboration with the National Centre for Voice and Speech, Denver, Colorado, who recorded the speech signals. The purpose of PD dataset is to discriminate healthy people from those with PD, given the results of various medical tests carried out on a patient. The PD dataset consists of voice measurements from 31 people of which 23 were diagnosed with PD. There are 195 instances comprising 48 healthy and 147 PD cases in the dataset. The time since diagnoses ranged from 0 to 28 years, and the ages of the subjects ranged from 46 to 85 years (mean 65.8). Each subject provides an average of six phonations of the vowel (yielding 195 samples in total), each 36 seconds in length [6]. Note that there are no missing values in the dataset and the whole features are real value. The whole 22 features along with description are listed in Table 1.

### 4.2. Experimental Setup

The proposed SCFW-KELM classification model was carried out on the platform of MATLAB 7.0. The SCFW algorithm was implemented from scratch. For KELM and ELM, the implementation available from http://www3.ntu.edu.sg/home/egbhuang/ was used.

For SVM, LIBSVM implementation was used, which was originally developed by Chang and Lin [27]. The empirical experiment was conducted on Intel Dual-Core TM (2.0 GHz CPU) with 2 GB of RAM.

In order to guarantee the valid results, *k*-fold CV was used to evaluate the classification results [28]. Each time, nine of ten subsets were put together to form a training set and the other subset was used as the test set. Then the average result across all 10 trials was calculated. Thanks to this method, all the test sets were independent and the reliability of the results could be improved. Because of the arbitrariness of partition of the dataset, the predicted results of model at each iteration were not necessarily the same. To evaluate accurately the performance of the PD dataset, the experiment was repeated 10 times and then the results were averaged.

### 4.3. Measure for Performance Evaluation

In order to evaluate the prediction performance of SCFW-KELM model, we used six performance metrics, ACC, sensitivity, specificity, AUC, *f*-measure, and kappa statistic value, to test the performance of the proposed model. About the mentioned performance evaluation formulations are defined as follows according to the confusion matrix which is shown in Table 2:

(9) $$\begin{array}{c} \text{ACC}=\frac{\text{TP}+\text{TN}}{\text{TP}+\text{FP}+\text{FN}+\text{TN}}\times 100\%,\\ \text{Sensitivity}=\frac{\text{TP}}{\text{TP}+\text{FN}}\times 100\%,\\ \text{Specificity}=\frac{\text{TN}}{\text{FP}+\text{TN}}\times 100\%,\\ \text{Precision}=\frac{\text{TP}}{\text{TP}+\text{FP}},\\ \text{Recall}=\frac{\text{TP}}{\text{TP}+\text{FN}},\\ f\text{-measure}=\frac{2\times \text{Precision}\times \text{Recall}}{\text{Precision}+\text{Recall}}. \end{array}$$

In the confusion matrix, TP is the number of true positives, which represents that some cases with PD class are correctly classified as PD. FN is the number of false negatives, which represents that some cases with the PD class are classified as healthy. TN is the number of true negatives, which represents that some cases with the healthy class are correctly classified as healthy and FP is the number of false positives, which represents that some cases with the healthy class are classified as PD. ACC is a widely used metric to determine class discrimination ability of classifiers. The receiver operating characteristic (ROC) curve is usually plotted using true positives rate versus false positives rate, as the discrimination threshold of classification algorithm is varied. The area under ROC curve (AUC) is widely used in classification studies with relevant acceptance and it is a good summary of the performance of the classifier [29]. Also *f*-measure is a measure of a test's accuracy, which is usually used as performance evaluation metric to assess the performance of binary classifier, based on the harmonic mean for the classifier's precision and recall. Kappa error (KE) or Cohen's kappa statistics (KS) is adopted to compare the performances of different classifiers. KS is a good measure to inspect classifications that may be due to chance. As KS value calculated for classifiers closer to 1, the performance of classifier is assumed to be more realistic rather than being by chance. Thus, KS value is a recommended metric to consider for evaluation in the performance analysis of classifiers and it is calculated with [30]

(10) $$\begin{array}{c} \text{KS}=\frac{P\left(A\right)-P\left(E\right)}{1-P\left(E\right)}, \end{array}$$

where *P*(*A*) means total agreement probability and *P*(*E*) means agreement probability due to chance.

## 5. Experimental Results and Discussions

**Experiment 1 (classification in the PD dataset).**

In this experiment, we firstly evaluated KELM in the original feature space without SCFW. It is known that different types of kernel activation functions have great influence on the performance of KELM. Therefore, we presented the results from our investigation on the influence of different types of kernel functions and assigned initial values for them. We tried to perform four types of kernel functions, including radial basis function (RBF_kernel), wavelet kernel function (Wav_kernel), linear kernel function (Lin_kernel), and polynomial kernel function (Poly_kernel).  Table 3 summarized the detailed results of classification performance in the PD dataset in terms of ACC, sensitivity, specificity, AUC, *f*-measure, and KS value, and these results were achieved via 10-fold CV scheme and represented in the form of average accuracy (Mean), standard deviation (SD), maximal accuracy (Max), and minimal accuracy (Min). From this table, it can be seen that the classification performance of KELM with various kernel functions is apparently differential. The best kernel function of KELM classifier in discriminating the PD dataset was RBF kernel function. We can see that KELM with RBF kernel outperforms that with the other three kernel functions with a mean accuracy of 95.89%, 96.35%, 95.72%, and 96.04% in terms of ACC, sensitivity, specificity, and AUC and has also got *f*-measure value of 0.9724 and KS value of 0.8925. KELM with wavelet kernel has obtained the average results of 94.36%, 91.24%, 95.25%, and 93.19% in terms of ACC, sensitivity, specificity, and AUC and got *f*-measure value of 0.9622 and KS value of 0.8425, lower than those of KELM with RBF kernel. The worse results of classification performance obtained by KELM with polynomial kernel and KELM with linear kernel were successively given. Noting training KELM with kernel functions instead of sigmoid additive function of ELM, the number of hidden neurons has no influence on the performance of KELM model, so it does not need to be considered.

**Figure 4:**
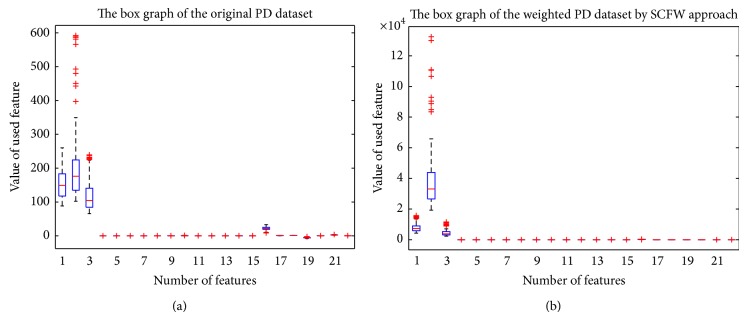
The box graph representation of the original and weighted PD dataset.

**Figure 5:**
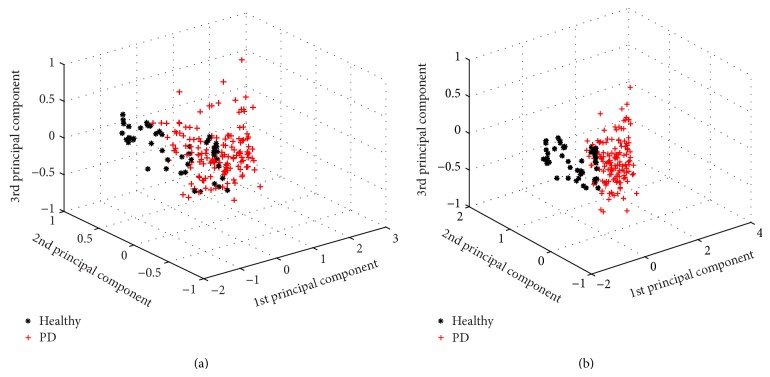
Three-dimensional distribution of two classes in the original and weighted feature space by the best three principle components obtained with PCA method.

To investigate whether SCFW method can improve the performance of KELM, we further conducted the model in the PD dataset in the weighted feature space by SCFW. The proposed system consisted of two stages. Firstly, SCFW approach was used to weight the features of PD dataset. By using SCFW method, the weighted feature space was constructed.  Table 4 listed the cluster centers of the features in the PD dataset using SCFW method.  Figure 4 depicted the box graph representation of the original and weighted PD dataset with the whole 22 features.  Figure 5 showed the distribution of two classes of the original and weighted 195 samples formed by the best three principle components obtained with the principle component analysis (PCA) algorithm [31]. From Figures 4 and 5, it can be seen that the discriminative ability of the original PD dataset has been improved substantially by SCFW approach. After data preprocessing stage, the classification algorithms have been used and discriminated the weighted PD dataset.

The detailed results obtained by SCFW-KELM with four types of different kernel functions were presented in Table 5. As seen from Table 5, all these best results were much higher than the ones obtained in the original feature space without SCFW. The classification performance in the PD dataset has significantly improved by using SCFW method. Compared with KELM with RBF kernel function in the original feature space, KELM with RBF kernel based on SCFW method increased the performance by 3.6%, 3.65%, 3.67%, and 3.65% in terms of ACC, sensitivity, specificity, and AUC and has obtained highest *f*-measure value of 0.9966 and highest KS value of 0.9863. The KELM models with the other three kernel functions also have got great improvements in terms of six performance metrics.

Table 6 also presented the comparison results of the confusion matrices obtained by SCFW-KELM and KELM. As seen from Table 6, SFCW-KELM correctly classified 194 normal cases out of 195 total normal cases and misclassified only one patient with PD as a healthy person, while KELM without SCFW method only correctly classified 187 normal cases out of 195 total normal cases and misclassified 6 patients with PD as healthy persons and 2 healthy persons as patients with PD.

For SVM classifier, we have performed SVM classifier with RBF kernel. It is known that the performance of SVM is sensitive to the combination of the penalty parameter *C* and the kernel parameter *γ*. Thus, the best combination of (*C*, *γ*) needs to select in the classification tasks. Instead of manually setting the parameters (*C*, *γ*) of SVM, the grid-search technique [32] was adopted using 10-fold CV to find out the best parameter values. The range of the related parameters *C* and *γ* was varied between *C* = [2^−15^, 2^−14^,…, 2^11^] and *γ* = [2^−15^, 2^−14^,…, 2^5^]. The combinations of (*C*, *γ*) were tried and the one with the best classification accuracy was chosen as the parameter values of RBF kernel for training model.

**Figure 6:**
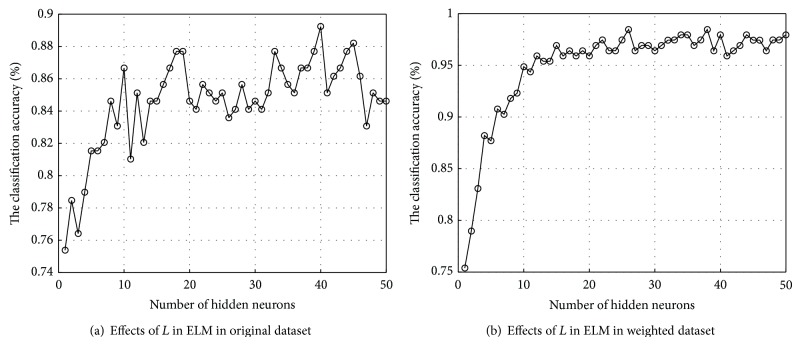
The effects of hidden neurons in original ELM in the classification of the original and weighted PD dataset.

For original ELM, we know that the classification performance of ELM with sigmoid additive function is sensitive to the number of hidden neurons *L*, so value of *L* needs to be specified by users.  Figure 6 presented the detailed results of ELM in the original and weighted PD dataset with different hidden neurons ranging from 1 to 50. Specifically, the average results of 10 runs of 10-fold CV for every specified neuron were recorded. As shown in Figure 6, the classification rates of ELM were improved with hidden neuron increasing at first and then gradually fluctuated. In the original dataset, it achieved highest mean classification accuracy with 40 hidden neurons, while in the weighted dataset with SCFW method, highest mean classification accuracy was gained with only 26 hidden neurons.

**Figure 7:**
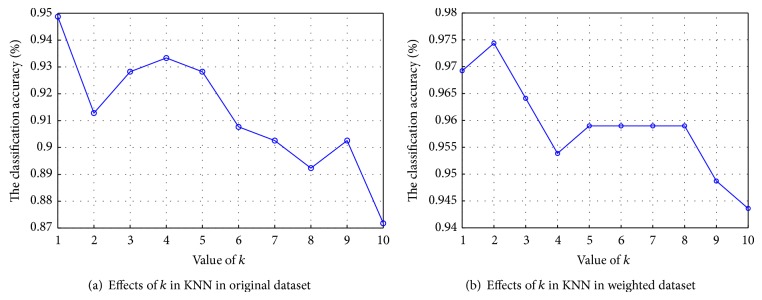
The effects of *k* in KNN in the classification of the original and weighted PD dataset.

For KNN classifier, the influence of neighborhood size *k* of KNN classifier in the classification performance of the PD dataset has been investigated. In this study, value of *k* increased from 1 to 10. The results obtained from KNN classifier with different values of *k* in the PD dataset are shown in Figure 7. From the figure, we can see that the best results have been obtained by 1-NN classifier, and the performance was decreased with the value of *k* increasing, while the better results were achieved in the weighted PD dataset with SCFW method for 2-NN.

**Figure 8:**
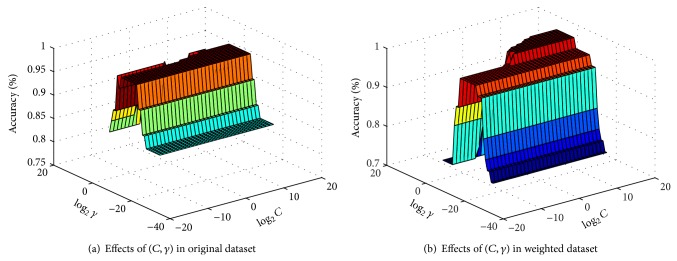
Test accuracy surface with parameters in KELM in the original and weighted PD dataset.

For KELM classifier, there were two parameters, the penalty parameter *C* and the kernel parameter *γ*, that need to be specified. In this study, we have conducted the experiments on KELM depending on the best combination of (*C*, *γ*) by grid-search strategy. The parameters *C* and *γ* were both varied in the range of [2^−15^, 2^−14^,…, 2^15^] with the step size of 1.  Figure 8 showed the classification accuracy surface in one run of 10-fold CV procedure, where *x*-axis and *y*-axis were log⁡_2_⁡*C* and log⁡_2_⁡*γ*, respectively. Each mesh node in the (*x*, *y*) plane of the classification accuracy represented a parameter combination and *z*-axis denoted the achieved test accuracy value with each parameter combination.

Table 7 summarized the comprehensive results achieved from four classifiers and those based on SCFW method in terms of ACC, sensitivity, specificity, AUC, *f*-measure, and KS value over 10 runs of 10-fold CV. Besides, the sum of computational time of training and that of testing in seconds was recorded. In this table, we can see that, with the aid of SCFW method, all these best results were much higher than the ones obtained in the original feature space. The SCFW-KELM model has achieved highest results of 99.49%, 100%, 99.39%, and 99.69% in terms of ACC, sensitivity, specificity, and AUC and got highest *f*-measure of 0.9966 and KS value of 0.9863, which outperforms the other three algorithms. Compared with KELM without SCFW, SCFW-KELM has improved the average performance by 3.6%, 3.65%, 3.67%, and 3.65% in terms of ACC, sensitivity, specificity, and AUC. Note that the running time of SCFW-KELM was extremely short, which costs only 0.0126 seconds.

In comparison with SVM, SCFW-SVM has achieved the results of 97.95%, 96.67%, 98.71%, and 97.6% in terms of ACC, sensitivity, specificity, and AUC and improved the performance by 2.57%, 11.58%, 0.04%, and 5.72%, respectively. KNN also has significantly improved by SCFW method. For ELM classifier, it has achieved best results by ELM with 36 hidden neurons on the original feature space, while the best performance was achieved by SCFW-ELM with small hidden neurons (only 26). It meant that the combination of SCFW and ELM not only significantly improved the performance but also compacted the network structure of ELM. Moreover, the sensitive results of SVM and ELM were significantly improved by 11.58% and 21.84%, respectively. Whatever in the original or weighted feature space, KELM with RBF kernel was much superior to the other three models by a large percentage in terms of ACC, sensitivity, specificity, AUC, *f*-measure, and KS value. Although SVM achieved the specificity of 98.67%, the sensitivity, AUC, *f*-measure, and KS value were lower than those of KELM with RBF kernel. We can also see that the performance of KELM with RBF kernel was much higher than those of ELM with sigmoid function. The reason may lie in the fact that the relation between class labels and features in the PD dataset is linearly nonseparable; kernel-based strategy works better for this case by transforming from linearly nonseparable to linearly separable dataset. However, the performances obtained by SCFW-SVM approach were close to those of SCFW-KNN. It meant that, after data preprocessing, SVM can achieve the same ability to discriminate the PD dataset as that of KNN.

Additionally, it is interesting to find that the standard deviation of SCFW-KELM was much lower than that of KELM, and it had the smallest SD in all of the models, which meant SCFW-KELM became more robust and reliable by means of SCFW method. In addition, the reason why SCFW method outperforms FCM is that SCFW may be more suitable for nonlinear separable datasets. It considers the density measure of data points to reduce the influence of outliers; however, FCM tends to select outliers as initial centers.

For comparison purpose, the classification accuracies achieved by previous methods which researched the PD diagnosis problem were presented in Table 8. As shown in the table, our developed method can obtain better classification results than all available methods proposed in previous studies.

**Experiment 2 (classification in two other benchmark datasets).**

Besides the PD dataset, two benchmark datasets, that is, Cleveland Heart and Wisconsin Diagnostic Breast Cancer (WDBC) datasets, from the UCI machine learning repository, have been used to further evaluate the efficiency and effectiveness of the proposed method. We used the same flow as in the PD dataset for the experiments of two datasets. The weighted features space of datasets was constructed using SCFW and then the weighted features were evaluated with the four mentioned algorithms. It will only give the classification results of four algorithms for the sake of convenience.  Table 9 showed the obtained results in the classification of the original and weighted Cleveland Heart dataset by SCFW-KELM model.  Table 10 presented the achieved results in the classification of the original and weighted WDBC dataset using SCFW-KELM model. As seen from these results, the proposed method also has achieved excellent results. It indicated the generality of the proposed method.

## 6. Conclusions and Future Work

In this work, we have developed a new hybrid diagnosis method for addressing the PD problem. The main novelty of this paper lies in the proposed approach; the combination of SCFW method and KELM with different types of kernel functions allows the detection of PD in an efficient and fast manner. Experiments results have demonstrated that the proposed system performed significantly well in discriminating the patients with PD and healthy ones. Meanwhile, the comparative results are conducted among KELM, SVM, KNN, and ELM. The experiment results have shown that the SCFW-KELM method performs advantageously over the other three methods in terms of ACC, sensitivity, specificity, AUC, *f*-measure, and kappa statistic value. In addition, the proposed system outperforms the existing methods proposed in the literature. Based on the empirical analysis, it indicates that the proposed method can be used as a promising alternative tool in medical decision making for PD diagnosis. The future investigation will pay much attention to evaluating the proposed method in other medical diagnosis problems.

## Figures and Tables

**Algorithm 1:**
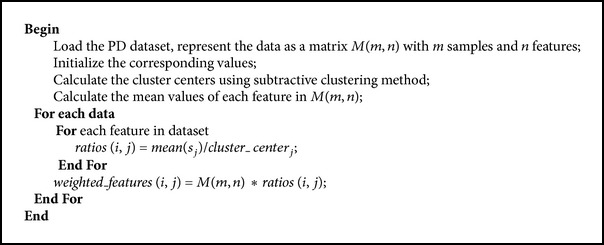
Pseudocode for weighting features based on subtractive clustering method.

**Algorithm 2:**
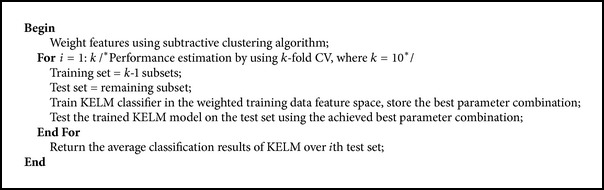
Pseudocode for the proposed model.

**Table 1:** The details of the whole 22 features of the PD dataset.

Label	Feature	Description
F1	MDVP: Fo (Hz)	Average vocal fundamental frequency
F2	MDVP: Fhi (Hz)	Maximum vocal fundamental frequency
F3	MDVP: Flo (Hz)	Minimum vocal fundamental frequency
F4	MDVP: Jitter (%)	Several measures of variation in fundamental frequency
F5	MDVP: Jitter (Abs)	
F6	MDVP: RAP	
F7	MDVP: PPQ	
F8	Jitter: PPQ	
F9	MDVP: Shimmer	Several measures of variation in amplitude
F10	MDVP: Shimmer (dB)	
F11	Shimmer: APQ3	
F12	Shimmer: APQ5	
F13	MDVP: APQ	
F14	Shimmer: DDA	
F15	NHR	Two measures of ratio of noise to tonal components in the voice
F16	HNR	
F17	RPDE	Two nonlinear dynamical complexity measures
F18	D2	
F19	DFA	Signal fractal scaling exponent
F20	Spread1	Three nonlinear measures of fundamental frequency variation
F21	Spread2	
F22	PPE	

**Table 2:** The confusion matrix.

	Predicted patients with PD	Predicted healthy persons
Actual patients with PD	True positive (TP)	False negative (FN)
Actual healthy persons	False positive (FP)	True negative (TN)

**Table 3:** Results of KELM with different types of kernel functions in the original PD dataset without SCFW.

Kernel type	Performance metrics	Mean	SD	Max	Min
RBF_kernel	ACC (%)	**95.89**	4.66	100	89.74
	Sensitivity (%)	**96.35**	5.19	100	88.89
	Specificity (%)	**95.72**	5.93	100	88.00
	AUC (%)	**96.04**	4.06	100	90.43
	*f*-measure	**0.9724**			
	Kappa	**0.8925**			

Wav_kernel	ACC (%)	94.36	4.59	100	87.18
	Sensitivity (%)	91.24	6.02	100	83.33
	Specificity (%)	95.15	5.23	100	86.21
	AUC (%)	93.19	4.56	100	88.10
	*f*-measure	0.9622			
	Kappa	0.8425			

Lin_kernel	ACC (%)	89.23	7.99	97.44	79.49
	Sensitivity (%)	66.07	22.33	90.91	41.67
	Specificity (%)	97.32	2.80	100	93.33
	AUC (%)	81.70	12.22	95.45	68.89
	*f*-measure	0.9316			
	kappa	0.6333			

Poly_kernel	ACC (%)	90.77	4.29	97.44	87.18
	Sensitivity (%)	87.73	11.54	100	75.00
	Specificity (%)	91.83	5.73	96.77	82.76
	AUC (%)	89.78	5.78	98.39	82.66
	*f*-measure	0.9375			
	kappa	0.7547			

**Table 4:** The cluster centers of the features of PD dataset using SCFW method.

Number of feature	Centers of the features using SCFW (normal case)	Centers of the features using SCFW (PD case)
F1	154.229	181.938
F2	197.105	223.637
F3	116.325	145.207
F4	0.006	0.006
F5	0	0
F6	0.003	0.003
F7	0.003	0.003
F8	0.01	0.01
F9	0.03	0.03
F10	0.282	0.276
F11	0.016	0.015
F12	0.018	0.018
F13	0.024	0.013
F14	0.047	0.045
F15	0.025	0.028
F16	21.886	24.678
F17	0.499	0.443
F18	0.718	0.696
F19	−5.684	−6.759
F20	0.227	0.161
F21	2.382	2.155
F22	0.207	0.123

**Table 5:** Results of SCFW-KELM with different types of kernel functions in the PD dataset.

Kernel type	Performance metrics	Mean	SD	Max	Min
RBF_kernel	ACC (%)	**99.49**	1.15	100	97.44
	Sensitivity (%)	**100**	0	100	100
	Specificity (%)	**99.39**	1.36	100	96.97
	AUC (%)	**99.69**	0.68	100	98.48
	*f*-measure	**0.9966**			
	Kappa	**0.9863**			

Wav_kernel	ACC (%)	96.92	2.15	100	94.87
	Sensitivity (%)	98.46	3.44	100	92.31
	Specificity (%)	96.54	2.39	100	93.33
	AUC (%)	97.50	2.18	100	94.23
	*f*-measure	0.9793			
	Kappa	0.9194			

Lin_kernel	ACC (%)	96.92	2.15	100	94.87
	Sensitivity (%)	90.43	8.85	100	81.82
	Specificity (%)	99.29	1.60	100	96.43
	AUC (%)	94.86	3.99	100	90.91
	*f*-measure	0.9798			
	Kappa	0.9147			

Poly_kernel	ACC (%)	97.43	2.56	100	94.87
	Sensitivity (%)	96.67	7.45	100	83.33
	Specificity (%)	97.37	3.61	100	93.10
	AUC (%)	97.02	3.42	100	91.67
	*f*-measure	0.9828			
	Kappa	0.9323			

**Table 6:** Confusion matrix of KELM with RBF kernel function in the original and weighted PD dataset.

Method	Expected output	Prediction output	
KELM	Patients with PD	141	6
	Healthy persons	2	46

SCFW-KELM	Patients with PD	146	1
	Healthy persons	0	48

**Table 7:** The results obtained from four algorithms in the original and weighted PD dataset.

Methods	Performance metrics	Original feature space without SCFW method	Weighted feature space with SCFW method
KELM-RBF	ACC (%)	95.89 ± 4.66	99.49 ± 1.15
	Sensitivity (%)	96.35 ± 5.19	100 ± 0
	Specificity (%)	95.72 ± 5.93	99.39 ± 1.36
	AUC (%)	96.04 ± 4.06	99.69 ± 0.68
	*f*-measure	0.9724	0.9966
	Kappa	0.8925	0.9863
	Time (s)	0.00435	0.0126

SVM	ACC (%)	95.38 ± 1.15	97.95 ± 2.15
	Sensitivity (%)	85.09 ± 10.45	96.67 ± 7.45
	Specificity (%)	98.67 ± 2.98	98.71 ± 1.77
	AUC (%)	91.88 ± 4.14	97.69 ± 3.46
	*f*-measure	0.9699	0.9863
	Kappa	0.8711	0.9447
	Time (s)	1.24486	1.29817

KNN	ACC (%)	95.38 ± 5.25	97.43 ± 3.14
	Sensitivity (%)	92.73 ± 11.85	97.78 ± 4.97
	Specificity (%)	96.50 ± 4.38	97.38 ± 4.10
	AUC (%)	94.61 ± 6.95	97.58 ± 2.60
	*f*-measure	0.9692	0.9828
	Kappa	0.8765	0.9431
	Time (s)	1.2847	1.3226

ELM	ACC (%)	89.23 ± 6.88	96.92 ± 4.21
	Sensitivity (%)	73.94 ± 13.18	95.78 ± 5.79
	Specificity (%)	93.35 ± 6.27	97.19 ± 4.51
	AUC (%)	83.64 ± 9.06	96.48 ± 4.36
	*f*-measure	83.64 ± 9.06	0.9863
	Kappa	0.7078	0.9447
	Time (s)	1.1437	1.2207

**Table 8:** Classification accuracies achieved with our method and other methods.

Study	Method	Accuracy (%)
Little et al. [6]	Preselection filter + exhaustive search + SVM	91.40 (bootstrap with 50 replicates)
Shahbaba and Neal [7]	Dirichlet process mixtures	87.70 (5-fold CV)
Das [8]	ANN	92.90 (hold out)
Sakar and Kursun [9]	Mutual information + SVM	92.75 (bootstrap with 50 replicates)
Psorakis et al. [10]	Improved mRVMs	89.47 (10-fold CV)
Guo et al. [11]	GP-EM	93.10 (10-fold CV)
Luukka [12]	Fuzzy entropy measures + similarity	85.03 (hold out)
Ozcift and Gulten [14]	CFS-RF	87.10 (10-fold CV)
Li et al. [13]	Fuzzy-based nonlinear transformation + SVM	93.47 (hold out)
Åström and Koker [15]	Parallel NN	91.20 (hold out)
Spadoto et al. [16]	PSO + OPF Harmony search + OPF Gravitational search + OPF	73.53 (hold out) 84.01 (hold out) 84.01 (hold out)
Daliri [19]	SVM with chi-square distance kernel	91.20 (50-50% training-testing)
Polat [17]	FCMFW + KNN	97.93 (50-50% training-testing)
Chen et al. [18]	PCA-FKNN	96.07 (average 10-fold CV)
Zuo et al. [20]	PSO-FKNN	97.47 (10-fold CV)
This study	SCFW-KELM	**99.49** (10-fold CV)

**Table 9:** Results of SCFW-KELM with different types of kernel functions in Cleveland heart dataset.

Kernel type	Performance metrics	Mean	SD	Max	Min
RBF_kernel	ACC (%)	**99.34**	0.91	100	98.33
	Sensitivity (%)	**100**	0	100	100
	Specificity (%)	**98.75**	1.72	100	96.67
	AUC (%)	**99.37**	0.86	100	98.33
	*f*-measure	**0.9964**			
	Kappa	**0.9867**			

Wav_kernel	ACC (%)	99.01	0.90	100	98.36
	Sensitivity (%)	100	0	100	100
	Specificity (%)	97.84	2.02	100	95.83
	AUC (%)	98.92	1.01	100	97.92
	*f*-measure	0.9891			
	Kappa	0.98			

Lin_kernel	ACC (%)	93.07	93.07	93.07	93.07
	Sensitivity (%)	98.77	98.77	98.77	98.77
	Specificity (%)	87.05	87.05	87.05	87.05
	AUC (%)	92.91	92.91	92.91	92.91
	*f*-measure	0.9195			
	Kappa	0.8591			

Poly_kernel	ACC (%)	98.35	2.33	100	95.08
	Sensitivity (%)	100	0	100	100
	Specificity (%)	96.60	5.01	100	88.89
	AUC (%)	98.30	2.50	100	94.44
	*f*-measure	0.9817			
	Kappa	0.9667			

**Table 10:** Results of SCFW-KELM with different types of kernel functions in WDBC dataset.

Kernel type	Performance metrics	Mean	SD	Max	Min
RBF_kernel	ACC (%)	**99.65**	0.79	100	98.23
	Sensitivity (%)	**99.05**	2.13	100	95.24
	Specificity (%)	**100**	0	100	100
	AUC (%)	**99.52**	1.06	100	97.62
	*f*-measure	**0.9972**			
	Kappa	**0.9925**			

Wav_kernel	ACC (%)	99.65	0.48	100	99.12
	Sensitivity (%)	99.10	1.24	100	97.62
	Specificity (%)	100	0	100	100
	AUC (%)	99.54	0.66	100	98.65
	*f*-measure	0.9958			
	Kappa	0.9925			

Lin_kernel	ACC (%)	98.07	1.69	100	95.61
	Sensitivity (%)	94.70	5.27	100	86.11
	Specificity (%)	100	0	100	100
	AUC (%)	97.35	2.63	100	93.06
	*f*-measure	0.9848			
	Kappa	0.9582			

Poly_kernel	ACC (%)	99.40	0.88	99.12	97.37
	Sensitivity (%)	95.33	2.07	97.73	93.48
	Specificity (%)	100	0	100	100
	AUC (%)	97.67	1.04	98.86	96.74
	*f*-measure	0.9944			
	Kappa	0.962			
